# Accessory right V^6^ behind the bronchus intermedius during VATS right upper lobectomy

**DOI:** 10.1016/j.ijscr.2019.02.014

**Published:** 2019-02-19

**Authors:** Dario Amore, Antonio Molino, Umberto Caterino, Carlo Bergaminelli, Dino Casazza, Albina Palma, Pasquale Imitazione, Carlo Curcio

**Affiliations:** a Thoracic Surgery Unit, Monaldi Hospital, Naples, Italy; b Department of Respiratory Diseases, Division of Pneumology, University of Naples Federico II, Monaldi Hospital, Naples, Italy; c Thoracic Endoscopic Unit, Monaldi Hospital, Naples, Italy; d Emergency Department, C.T.O., Naples, Italy

**Keywords:** Accessory right V^6^, Vascular anomaly, VATS lobectomy

## Abstract

**Introduction:**

The anatomical abnormalities in pulmonary veins can have a serious impact on pulmonary resections.

**Presentation of case:**

We report the case of a 70-year-old woman undergoing VATS right upper lobectomy for the treatment of non-small cell lung cancer. During subcarinal dissection, an anomalous vein draining from the superior segment of the right lower lobe into the left atrium and passing behind the bronchus intermedius was incidentally discovered. The patient had, in addition to the inferior pulmonary vein formed by the confluence of superior and common basal veins, a supernumerary vessel identified as: accessory right V^6^. Retrospective review of preoperative enhanced chest computed tomography confirmed the pulmonary vascular anomaly.

**Discussion/Conclusion:**

A careful dissection during pulmonary resections can help to recognize variations of the pulmonary veins, avoiding unexpected intraoperative complications.

## Highlights

• The drainage pattern of the pulmonary veins presents a wide range of anatomical variations.
• Identification of anatomical variations in pulmonary veins can help avoid intraoperative complications.
• A careful dissection during VATS lobectomy is essential to avoid intraoperative complications.

## 1 Introduction

The anatomical abnormalities in pulmonary veins have always aroused great interest among the cardiothoracic surgeons, radiologists, clinical anatomists and in literature, over the years, numerous variations of pulmonary veins have been reported [[1], [2], [3], [4]]. The advent of VATS (video-assisted thoracic surgery), adopted with such enthusiasm also for major lung resections, has revived interest in these vascular anomalies that can lead to serious unexpected bleeding during surgery when not recognized [3,5,6]. Minimally invasive approaches in thoracic surgery have been widely appreciated thanks to well documented early postoperative benefits but some limits, such as reduced tactile sensation and restricted range of motion [7], could be an obstacle to safe surgical dissection in presence of vascular variations missed preoperatively. Reported causes of conversion to thoracotomy during VATS lobectomy are not only fused interlobar fissure, calcified hilar adenopathy, oncologic problems but also vascular anomalies [8,9]. A careful evaluation of anatomical structures is therefore essential in thoracic surgery to perform safe pulmonary resections and the role of imaging studies is particularly useful in predicting surgical complications associated with VATS [10].

## 2 Presentation of case

**Fig. 1:**
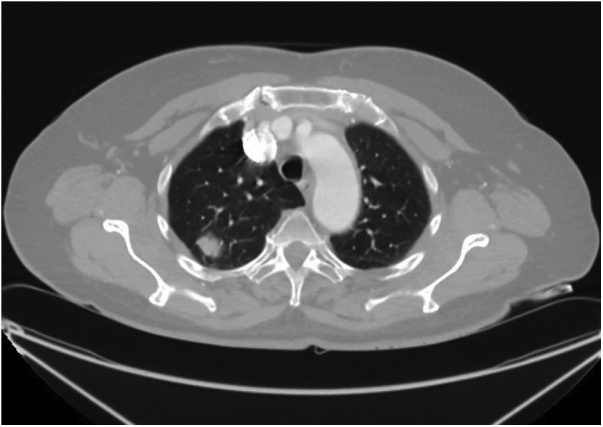
Chest CT image showing malignant lesion in the apical segment of the right upper lobe.

**Fig. 2:**
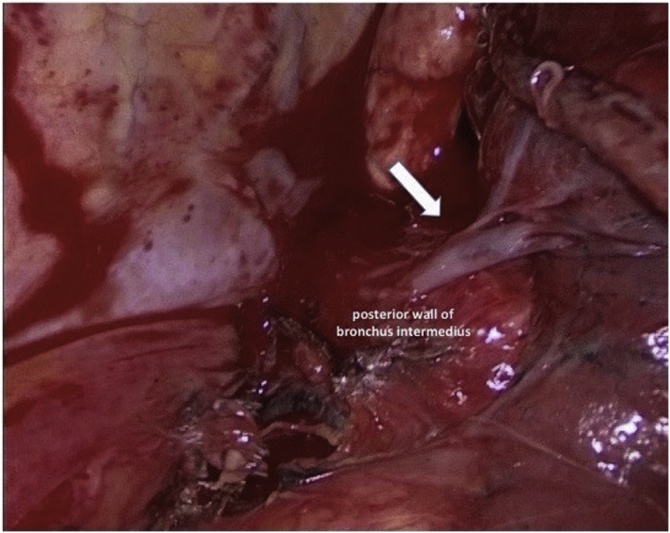
Intraoperative findings show an anomalous vein behind the bronchus intermedius during subcarinal dissection (white arrow).

**Fig. 3:**
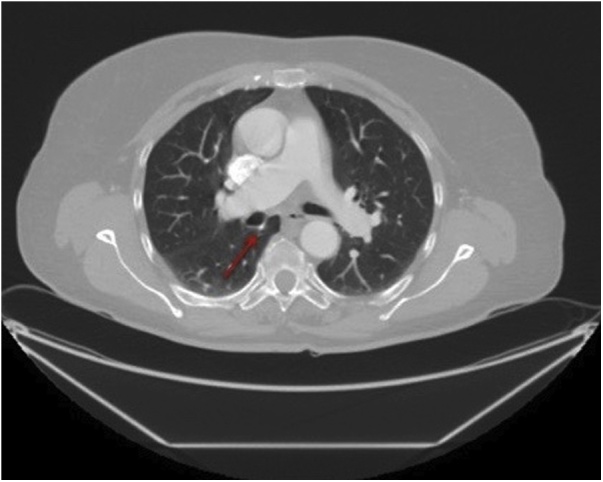
Retrospective review of chest CT shows a venous branch (red arrow) behind the bronchus intermedius.

**Fig. 4:**
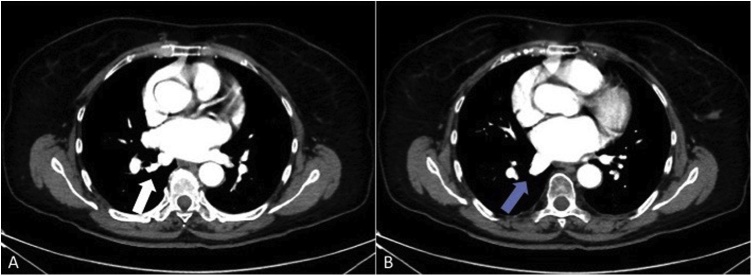
(A and B) Mediastinal window of chest CT. The radiologic images show the right inferior pulmonary vein formed by the hilar union of superior (white arrow) (A) and common basal veins (blue arrow) (B) from the lower lobe.

A 70-year-old woman with a positive cytologic diagnosis of lung adenocarcinoma, accomplished by fine needle aspiration biopsy (FNAB), was admitted to our unit. A preoperative enhanced chest computed tomography (CT) revealed a lesion, measuring 20 × 19 mm, in the right upper lobe apical segment (Fig. 1). Positron-emission tomography (PET) demonstrated intense focal fluorodeoxyglucose uptake in the pulmonary nodule with a standardized uptake value of 6,7. The patient, preoperatively staged as cT1bN0M0, underwent VATS right upper lobectomy and systematic lymphadenectomy performed through a three-port anterior approach and using a double-lumen endobronchial tube for one-lung ventilation. After dissecting and dividing the pulmonary vein, arteries and bronchus of the upper lobe, the posterior aspect of the hilum was exposed for mediastinal lymphadenectomy. An anomalous vein from superior segment of right lower lobe, draining directly into the left atrium, was discovered behind the bronchus intermedius (Fig. 2). A careful mediastinal lymph node dissection allowed to preserve it and the patient had an uneventful postoperative course. Retrospective review of the preoperative chest CT showed a focal nodularity in the posterior wall of the bronchus intermedius (Fig. 3). This nodularity was found to correspond to a venous branch draining from the right upper segment of the lower lobe into the left atrium (Video 1). The supernumerary vessel was identified as accessory V^6^ because chest CT showed a right inferior pulmonary vein typically formed by the union of the superior vein (V^6^) and the common basal vein (Fig. 4A and B).

## 3 Discussion

Anatomical variations of pulmonary veins are more common than those of pulmonary arterial branches and are more frequently related to right pulmonary venous drainage patterns [2,11]. Many vascular anomalies have been described in literature and, among them, some authors have reported cases of a vein passing posterior to the bronchus intermedius: this anatomical variation almost always concerned the posterior segmental vein of right upper lobe draining directly into the left atrium, into the right superior pulmonary vein or into the right inferior pulmonary vein [3,12,13]. Kim et al. described a vein from the superior segment of the right lower lobe appearing, on chest CT scans, as a nodular opacity in the posterior wall of the bronchus intermedius but in all patients it drained into the right inferior pulmonary vein [13]. In our case the anomalous venous branch, behind the bronchus intermedius, drained from the superior segment of the right lower lobe directly into the left atrium. The importance of such anatomical variation is based on the fact that it represents a possible cause of haemorrhage when not recognized during subcarinal dissection. If anatomical abnormalities in pulmonary veins are overlooked, life-threatening complications such as uncontrolled bleeding, severe lung edema, may occur during pulmonary resections including hilar dissection, fissure dissection or mediastinal and interlobar lymphadenectomy [12]. Preoperative and intraoperative identification of pulmonary vascular anomalies is essential to reduce risk of complications especially in minimally invasive thoracic surgery where the surgical field, more limited than in thoracotomy, can make hemostasis following vessel injury more difficult to achieve [3]. During surgical resections in VATS, only an adequate and careful dissection can help prevent potential morbidity and mortality. We state that this work has been reported in line with the SCARE criteria [14].

## 4 Conclusion

Preoperative identification of anatomical variations in the pulmonary venous system is the right solution to prevent intraoperative complications during pulmonary surgery. However, some vascular anomalies are recognized only on retrospective review of imaging studies and therefore, during surgical procedures, a careful dissection is essential to identify unexpected anatomical abnormalities in pulmonary veins. To our knowledge, accessory right V^6^ passing behind the bronchus intermedius has never been discovered during VATS lobectomy.

## Conflicts of interest

There is no conflict of interest for any of the authors.

## Sources of funding

The authors state that the case report was produced in the absence of economic funding sources.

## Ethical approval

Ethical approval was not required from my Institution for this case report.

## Consent

Written informed consent was obtained from the patient for publication of this case report and accompanying images. A copy of the written consent is available for review by Editor-in-Chief of this journal on request.

## Author’s contribution

Dario Amore conceptualised the study, performed a literature review and drafted the manuscript.

Umberto Caterino and Carlo Bergaminelli performed a literature review and drafted the manuscript.

Dino Casazza, Albina Palma and Pasquale Imitazione performed a literature review and collected data.

Carlo Curcio and Antonio Molino critically revised the article.

All authors approved submission of the final article.

## Registration of research studies

Not applicable.

## Guarantor

Dario Amore, MD.

## Provenance and peer review

Not commissioned, externally peer-reviewed.
