# Supplementary material for: Accessory right V6 behind the bronchus intermedius during VATS right upper lobectomy
Source: Int J Surg Case Rep. 2019 Feb 19;56:17–9. doi: 10.1016/j.ijscr.2019.02.014 (PMC6389542; doi:10.1016/j.ijscr.2019.02.014)
Supplement: Supplementary file 1 [file mmc1.docx]

**Video 1**

Preoperative chest CT shows the right common basal vein, the venous branch from the superior segment of right lower lobe (right V^6^) and another venous branch, behind the bronchus intermedius, arising from the superior segment of right lower lobe and draining into the left atrium (accessory right V^6^).
